# Chemical, Biological and Morphological Properties of Fine Particles during Local Rice Straw Burning Activities

**DOI:** 10.3390/ijerph18158192

**Published:** 2021-08-02

**Authors:** Nur Amanina Ramli, Noor Faizah Fitri Md Yusof, Kamarul Zaman Zarkasi, Azrin Suroto

**Affiliations:** 1 School of Civil Engineering, Engineering Campus, Universiti Sains Malaysia, Nibong Tebal 14300, Pulau, Malaysia; amaninaramli19.msc@student.usm.my (N.A.R.); azrinsuroto@student.usm.my (A.S.); 2 Microbial Ecology and Bacteriology Laboratory, School of Biological Sciences, Universiti Sains Malaysia, Minden 11800, Pulau, Malaysia; kamarul.zarkasi@usm.my

**Keywords:** air pollution, rice straw burning, PM_2.5_, morphological properties, chemical properties, biological compositions

## Abstract

Rice straw is commonly burned openly after harvesting in Malaysia and many other Asian countries where rice is the main crop. This operation emits a significant amount of air pollution, which can have severe consequences for indoor air quality, public health, and climate change. Therefore, this study focuses on determining the compositions of trace elements and the morphological properties of fine particles. Furthermore, the species of bacteria found in bioaerosol from rice burning activities were discovered in this study. For morphological observation of fine particles, FESEM-EDX was used in this study. Two main categories of particles were found, which were natural particles and anthropogenic particles. The zinc element was found during the morphological observation and was assumed to come from the fertilizer used by the farmers. ICP-OES identifies the concentration of trace elements in the fine particle samples. A cultured method was used in this study by using nutrient agar. From this study, several bacteria were identified: *Exiguobavterium indicum, Bacillus amyloliquefaciens, Desulfonema limicola str. Jadabusan, Exiguobacterium acetylicum, Lysinibacillus macrolides,* and *Bacillus proteolyticus*. This study is important, especially for human health, and further research on the biological composition of aerosols should be conducted to understand the effect of microorganisms on human health.

## 1. Introduction

The combustion of living or dead plants, such as grassland, forest, and agricultural waste, as well as biomass for fuel, is referred to as biomass burning. Physical and chemical reactions, as well as mass and heat transfer, are all involved in this complex process [1]. Land cover changes were taken into account when categorizing biomass burning into four types: crop residue burning, shrubland fires, forest fires, other fires [2]. Biomass burning in agricultural fields is practiced to remove residues after the harvest and to control weeds and release nutrients for the next crop cycle, while in forests it can contribute to agricultural clearing and assist with the collection of food products [3,4]. Open-field burning has many advantages, such as being effective, reliable, and inexpensive [5]. This low cost method of crop residue disposal is used in many parts of the world to clear land of surface biomass to facilitate crop rotation, to control undesirable weeds, pests and diseases, and to replenish the soil with nutrients [6].

The field burning of rice straw is commonly practiced in the region when there is a short duration to prepare the field for the next crop [7]. There are different practices for rice straw burning (e.g., pile burning or burning of straw that is consistently spread over the field). This activity releases a large amount of air pollutants, which can cause serious effects on ambient air quality, public health, and climate [8]. The incomplete combustion of rice straw in the field emits a considerable amount of contaminants, including poisonous gases (carbon monoxide (CO), volatile organic compounds (VOC), and carcinogenic polycyclic aromatic hydrocarbons) and fine/inhalable particles. Ineffective straw management, such as open field burning, leaving it on the field, and dumping it in landfills, degrades the atmosphere and has a negative impact on public health.

**Figure 1:**
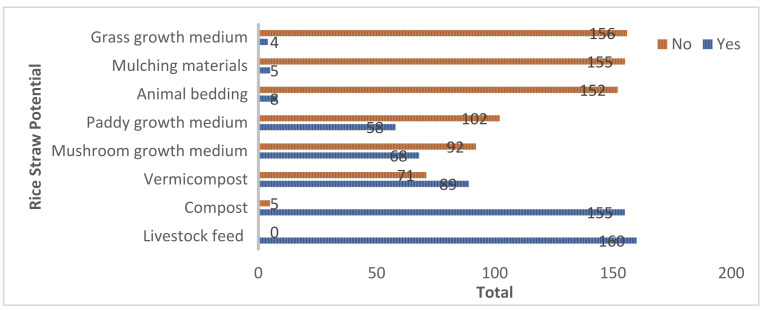
The farmer’s knowledge towards rice straw potential in the agricultural sector [9].

A study has been conducted by interviewing farmers about the awareness of rice straws in the agricultural sector. The findings indicate that most farmers are unaware of how to use straw in agricultural activities. According to the interviews, reusing straw in agricultural activities will improve soil quality, improve plant growth, improve livestock and seedling germination, increase yield production, and reduce farm management costs [9]. The result shows in Figure 1 that although there are numerous applications in agriculture, only a small percentage of farmers from 160 correspondents are taking advantage of its importance and potential.

Bioaerosols are biologically derived suspended airborne particles that are widely dispersed (bacteria, viruses, fungi, toxins, pollen, etc.) [10,11]. There are various tiny species in the air, ranging in size from 50 to 10 µm. These species are known as bioaerosols or airborne biological particles. The recognition of bioaerosols and the creation of control methods against them have played critical roles in the human history of researching the origins of life and combating infectious diseases [10]. Bioaerosol is primarily generated by interregional transport, biomass combustion, and soil and plant dust [12].

The aim of this study is to establish a causal connection between rice straw open burning and ambient air quality in Bagan Serai, Perak, Malaysia. The chemical, biological, and morphological properties of fine particles from the emissions and the potential impacts on the air quality in the neighboring areas were investigated in this study. Furthermore, the importance of studying bioaerosols and determining their relation to health and climate appears to be critical.

## 2. Materials and Methods

### 2.1. Concentration of PM_2.5_ and Meteorological Data and Sample Collection

**Figure 2:**
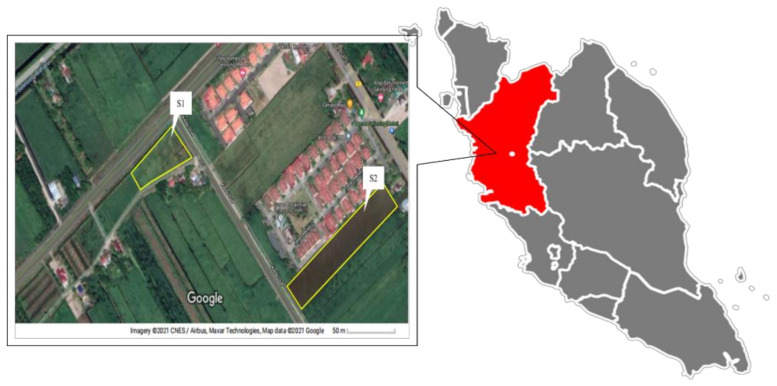
Site Sampling at Bagan Serai, Perak, Malaysia.

Monitoring was carried out for 12 h between 1 October 2020 and 28 October 2020 starting from 7.00 a.m. until 7.00 p.m. Two sites were selected and at each sampling point, 3 days of monitoring was conducted (1 day for biomass burning activities and 2 days for background sampling). On 1 October 2020, sampling activity during biomass burning activities was performed at S1, and sampling for background was conducted on 27 and 28 October 2020 after biomass burning events. S2 sampling occurred during biomass burning activities on 3 October 2020, and background sampling occurred before and after the day of biomass burning activities on 2 and 4 October 2020. Both sites are located at Bagan Serai in Perak, Malaysia; S1 (4°56′00.6″ N, 100°38′20.0″ E) and S2 (4°55′59.2″ N, 100°38′33.0″ E), as shown in Figure 2. The mass concentration of PM_2.5_ was determined using E-BAM (Met One Instrument Inc., Grants Pass, OR, USA), which employs the beta attenuation method. Samples of fine particles were collected on glass-fiber filter tape with a pore size of 2 µm using same equipment. Aside from fine particles, E-BAM constantly monitors meteorological parameters such as ambient temperature (°C), wind speed (m/s), and relative humidity (%) at 1 min intervals and 16.7 Lpm flow rate. The particles accumulated in 11 mm diameter spots on glass fiber filter tape, and the tapes advanced automatically every hour, forming one spot per hour. PM_2.5_ samples were collected continuously for 12 h (1–12 h). The samples were immediately transferred to a petri dish and refrigerated (<4 °C) until further analysis.

### 2.2. Physicochemical Analysis of Ambient PM_2.5_ Samples

Fine particle (PM_2.5_) samples were collected on fiberglass filter tape and analyzed for morphology, size of particles, and elemental compositions of individual particles using high-resolution field emission scanning electron microscopy coupled with energy dispersive X-ray (FESEM-EDX) (Quanta FEG 650, Oxford Instrument, Abingdon, UK). Various samples were chosen and analyzed. A steel puncher with a diameter of 12 mm was used to punch each spot of samples from the glass fiber tape. As the second half of the sample was needed for additional analysis, the sample was cut in two with a disposable scalpel. A carbon tape-wrapped stub was used to mount the sample, which was then coated with a thin sheet of gold using a coater (Quorum 150T). To determine the morphological properties and elemental compositions of particles, the coated samples were manually examined using FESEM-EDX at magnifications 10,000×. The technique of energy dispersive X-ray (EDX) was used to determine the elemental compositions of the samples. The EDX analysis generated data in the form of spectra with peaks corresponding to the elements that comprise the true composition of the sample, and the results also revealed the elemental composition content in form of weight percentage.

### 2.3. Collection of Culturable Airborne Bacteria

Microbial sampling was performed using Bio-Culture™ Model B30120 to sample the culturable airborne bacteria at a flow rate of 120 LPM. An agar dish containing nutrient agar was placed and positioned within the mount of the instrument. Airborne particles were allowed to settle onto the plate for 5 min and the plates were then closed. The airborne bacteria collection plates were then cultured for 48 h at 37 °C.

#### 2.3.1. Plating and Enrichment

The process entailed collecting bioaerosol samples in a specific working environment and cultivating them on agar nutrient media. The morphologically distinct grown colonies were then chosen, picked, and a single type of bacteria isolated from the mixture of bacteria. The streak method was used to isolate a single type of bacteria. Individual cells were diluted by spreading them over the surface of a new agar plate and incubating them again. Following incubation, a single colony was selected and transferred into DNA-free reaction tubes individually [13].

#### 2.3.2. DNA Barcoding

GoTaq Green Master Mix was used to mix a single colony. Reaction mixes for 25 µL reaction volume were prepared in accordance with the manufacturer’s instructions. PCR was operated on individual bacterial colonies using Applied Biosystems^®^ Veriti^®^ 96-Well Thermal Cycler (ThermoFisher SCIENTIFIC, Waltham, Massachusetts, United States of America). Polymerase chain reaction (PCR) was performed for exponentially amplifying target sequences from a DNA template. For bacterial DNA amplification, 27F (5′-AGAGTTTGATCCTGGCTCAG-3′) and 1492R (5′-TACGGYTACCTTGTTACGACTT-3′) primers were used. Denaturation at 94 °C for 10 min, 35 cycles of 94 °C for 30 s, 55 °C for 30 s, and 72 °C for 1 min 50 s, and final elongation at 72 °C for 7 min were used in the PCR. The amplified fragments were isolated from the gel and purified with a QIAquick PCR Purification Kit (QIAGEN). Purified PCR products were sequenced on an Applied Biosystems 3500 sequencer with a BigDye Terminator v3.1 Cycle Sequencing Kit and the obtained nucleotide sequences were finally aligned against the NCBI database for bacterial identification. The BlastN program was used to approximate taxonomic identification of sequences obtained from 16S rDNA of endophytic bacterial isolates [14]. This method was used for a total of six culturable isolates from PM_2.5_ samples.

## 3. Results

### 3.1. The Concentration of PM_2.5_ during Rice Straw Burning Activities

**Figure 3:**
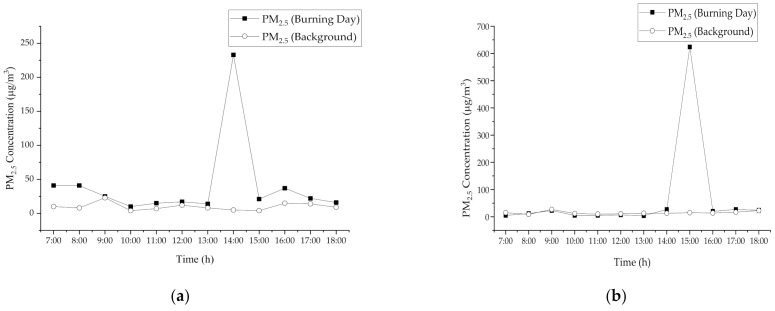
Concentration of ambient PM*_2.5_* during rice straw burning activity (**a**) Site 1 (**b**) Site 2.

Figure 3 shows the diurnal variations during burning activities and during non-burning activities for both sites (a) S1 and (b) S2. The patterns of hourly changes in PM_2.5_ concentrations are based on this figure, i.e., the trends show that the emission of fine particles during rice straw burning activities were similar. The result shows that the 12 h mean average concentration ± standard deviation of PM_2.5_ S1 (BB) (41.00 ± 58.82 µg m^−3^) and at S2 (BB) (65.33 ± 175.10 µg m^−3^) exceeded the limit suggested by MAAQS-2020 and USEPA, which is 35 µg m^−3^. The burning activities at S1 occurred from 13:00 p.m. to 14:00 p.m. and for site 2, the combustion activities started at 14:00 p.m. to 15:00 p.m. Based on the results, the concentration of PM_2.5_ peaks during the rice straw burning activities and only lasts for an hour. This finding indicates that the emissions of fine particles from agricultural burning activities have not taken place for a long time. This proved the statement reported by Tipayarom and Oanh (2007) that short-term and intensive emission practices such as biomass burning are most likely to blame for the extremely fluctuating air pollution levels from day to day [8].

**Figure 4:**
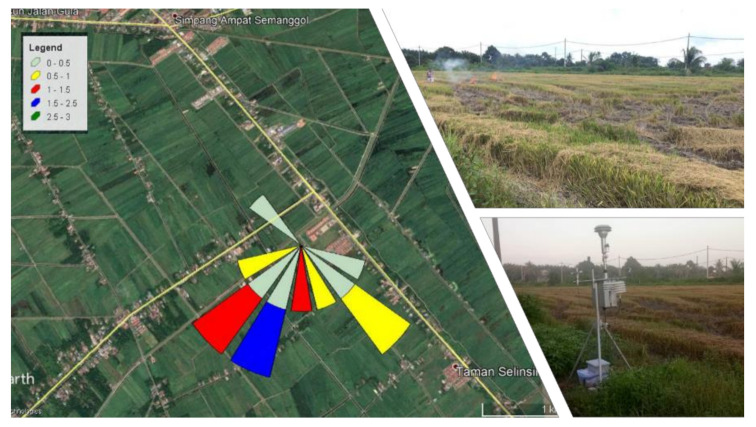
Wind rose plot, blowing from 12 h at Site 1.

**Figure 5:**
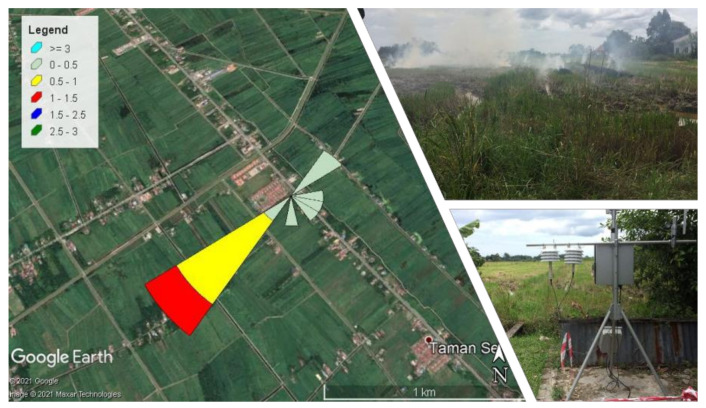
Wind rose plot, blowing from 12 h at Site 2.

The wind rose diagram was built using hourly wind data. Figure 4 and Figure 5 depict the wind direction at the sampling area. The result shows the frequency of the wind and transportation of the rice straw burning emissions directly to the station based on the wind rose plot on both sides, with the highest wind speeds of 3.30 m/s at S1 and 2.50 m/s at S2. These confirmed the possible sources that contributed to the high PM_2.5_ concentration at S1 and S2 was from the rice straw burning event.

### 3.2. The influence of Meteorological Conditions on PM_2.5_ Concentration

Local pollution emissions, external pollution transmission, and meteorological conditions such as pressure, temperature, humidity, cloud coverage, precipitation, and wind all affect PM_2.5_ concentrations. Meteorological factors influence the conglomeration and diffusion of pollutants, causing spatiotemporal variation in particulate matter concentrations [15,16].

The overall mean of PM_2.5_ concentration and meteorological parameters for both sites during two conditions, which were during background (non-burning activities) and during biomass burning. Background sampling activities were conducted at S1, days after the biomass burning events, and at S2 before and after biomass burning events, which are summarized in Table 1. The mean PM_2.5_ concentration for background (B) and biomass burning (BB) showed the highest value during rice straw burning activities compared with during non-burning activities for both sites S1 and S2. The highest mean measured was 65.33 µg/m^3^ at S2 during biomass burning activity. The median PM_2.5_ concentration ranged from 9.00 to 21.50 µg/m^3^. The average relative humidity and wind speed were in the range of 60.59% to 67.95% and 0.34 to 0.81 m/s, while the average temperature for these two sites was in the range of 28.88 to 31.75 °C for both sites.

**Figure 6:**
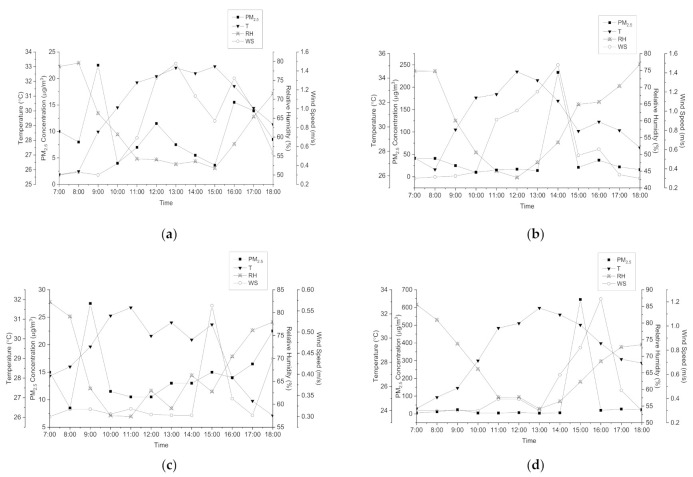
Diurnal plot of PM_2.5_ concentration and meteorological parameters at (**a**) S1 (B), (**b**) S1 (BB), (**c**) S2 (B), and (**d**) S2 (BB).

The changes in PM_2.5_ concentration with meteorological parameter patterns in two different locations with two different conditions with time are presented in Figure 6. The diurnal plot of temperature for both sites during burning activities and non-burning activities showed that as the air temperature increased, the relative humidity significantly decreased. Based on Figure 6, PM_2.5_ concentration showed an inversely proportional diurnal trend with wind speed and temperature, while a proportional trend was shown between the concentration of fine particles and relative humidity at both sites during burning activities and non-burning activities. According to Yin et al. 2016, the PM concentration was obviously high when the wind speed was low (less than 4 m/s). Furthermore, as wind speed (4–8 m/s) increased, PM concentration decreased [17]. This suggests that while low wind speeds may restrict particulate distribution, higher wind speeds may restrain particulate accumulation and therefore lower PM_2.5_ pollutant concentrations. However, as the wind speed began to increase, the PM concentration rose suddenly. This phenomenon may have been be caused by pollutants brought by strong winds from nearby areas. The maximum relative humidity occurred at S2 during the background monitoring campaign, as it was raining during the sampling period. The PM_2.5_ concentration, however, increased as the relative humidity rose. These results were found to be consistent with the previous studies [18,19].

### 3.3. Morphological Properties of PM_2.5_ during Rice Straw Burning Activities

**Figure 7:**
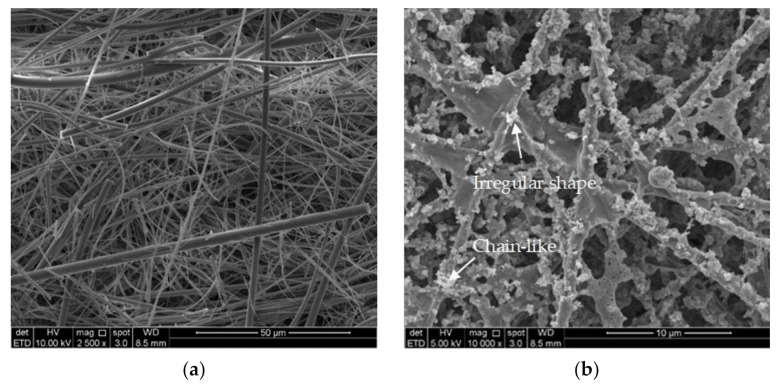
Micrograph of (**a**) blank glass-fiber filter paper and (**b**) glass-fiber filter paper spots that contained PM_2.5_ samples.

The micrograph of blank glass-fiber filter paper and glass-fiber filter paper spots that contained PM_2.5_ samples analyzed by FESEM-EDX are shown in Figure 7a,b respectively. Based on the morphological analysis of PM_2.5_ conducted, the particles collected on the glass fiber filter had a chain-like and irregular shape. The particles varied in size but were not larger than 2.5 µm.

**Figure 8:**
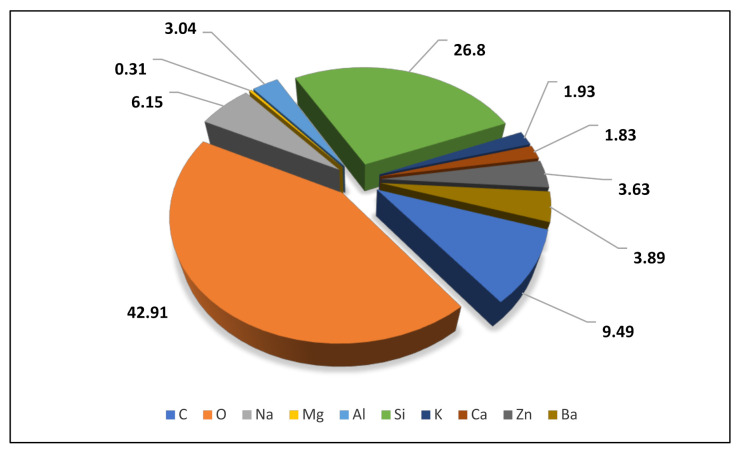
Weight percentage of each element found in glass-fiber filter paper from EDX analysis for blank sample.

From the EDX analysis of a blank sample, Figure 8 indicates the weight percentage of each element found in the glass fiber filter paper from the EDX analysis for blank sample. The elements found in the blank sample were C, O, Na, Mg, Al, Si, K, Ca, Zn, and Ba.

**Figure 9:**
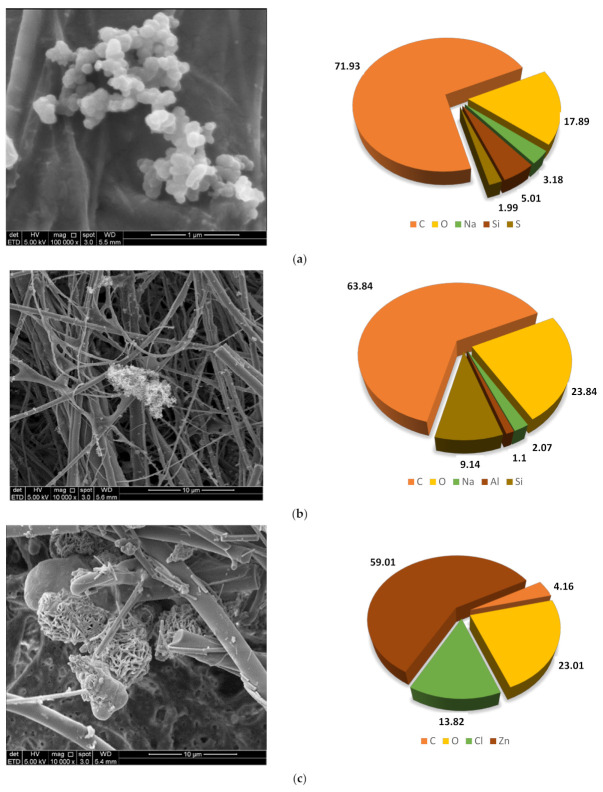
(**a**) Natural particles; (**b**) anthropogenic particles; (**c**) major zinc element in PM2.5 samples.

Three glass-fiber filter paper spots that contained PM_2.5_ samples were analyzed by FESEM-EDX to obtain the structure, particle size, and morphological features. The result revealed two major categories: natural sources and anthropogenic sources. Anthropogenic sources include metals, fly ash, soot, and organic particles, while natural sources include minerals or soil dust or minerals. Several studies have classified natural particle sources into three categories: anthropogenic windblown dust from human-disturbed soils due to improvements in land use practices, cultivation, and deforestation, which are mainly emitted from high-temperature combustion processes [20,21]. Soot is a clump of many fine spherical primary particles. Figure 9a,b shows major components of C, O, and Si, which are considered as biomass burning soot produced by agriculture activities. It also has an irregular morphology of various shapes. From Figure 9c, the highest component of Zinc was formed in this aggregate. With the addition of zinc, grain and straw yields in various rice genotypes increased by 14 and 16 percent, respectively [22]. Thus, this proved that the Zn element came from the fertilizer used by the farmers to keep maintaining the productivity of the rice production.

### 3.4. Variations of PM_2.5_ Trace Elements

**Figure 10:**
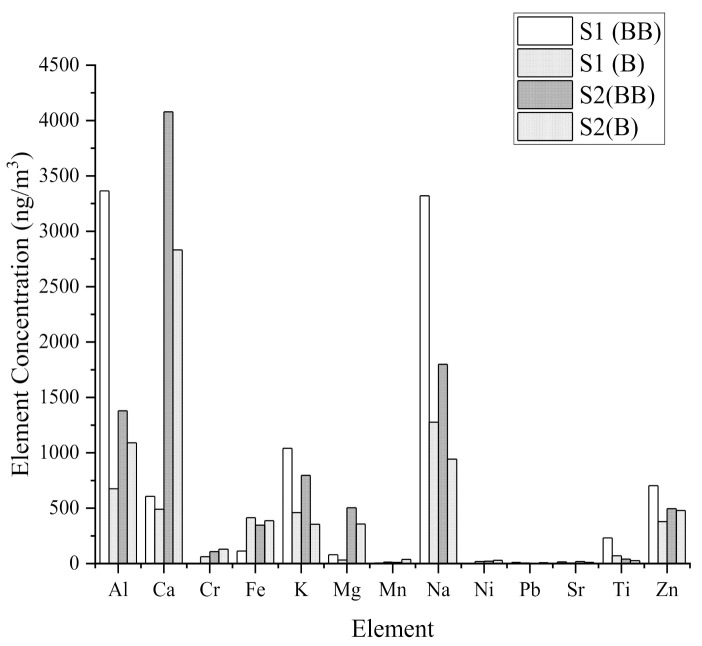
Average elemental concentration at Site 1 and Site 2 during background and biomass burning activities.

Based on Figure 10, thirteen elements were analyzed including Al, Ca, Cr, Cu, Fe, Pb, Mg, Mn, Ni, K, Na, Sr, Ti, and Zn in the field of rice straw burning. The levels of PM_2.5_ chemical component concentration in Figure 10 showed different patterns during biomass burning activities and during non-biomass burning activities. Ca, Al, Na, K, and Zn element concentrations were found to be higher on average than other trace elements. The Al was highest for both sites during rice straw burning activities, at 3364 ng/m^3^ and 1379 ng/m^3^. Ca showed the highest concentration at S2 during burning activities at 3320 ng/m^3^.

### 3.5. Microbiol Composition Analysis

**Figure 11:**
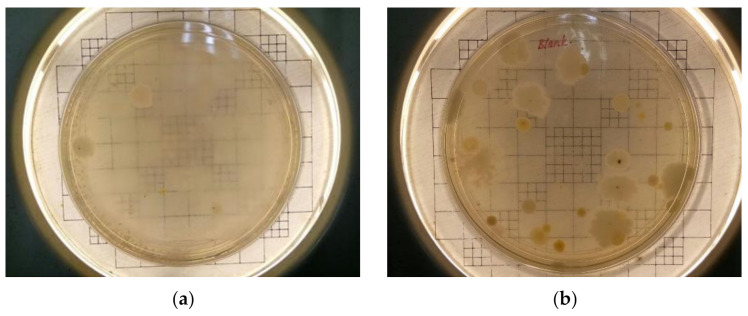

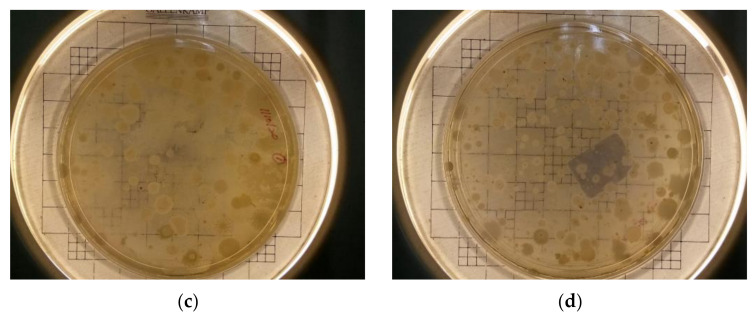
Samples collection after incubation period for blank (gravitational method) from Site 1 (**a**) and Site 2 (**b**) and samples collected using Bio-Culture™ Model B30120 for 5 min at Site 1 (**c**) and Site 2 (**d**) during Rice Straw Burning Activities.

**Figure 12:**
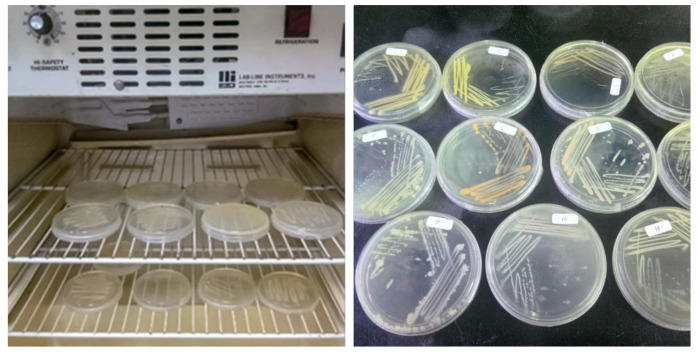
Isolated bacteria were streaked on new plates of nutrient agar and incubates at 37 °C for another 48 h.

Bioaerosol samples were plated on nutrient agar medium, and the grown colonies were used for Sanger sequencing of the 16s rDNA gene fragment. The sample was incubated at 37 °C for 48 h and the colonies were observed, as shown in Figure 11. The colonies formed on the agar plate were picked based on the color and shapes and streaked on the new agar plate and incubated at 37 °C for another 48 h as shown in Figure 12.

#### Bacteria Identification

The bacteria identified in the airborne sample are listed in the table based on the sanger sequencing results as shown in Table 2. However, not all isolated bacteria strains were identified in this study, with the Sanger sequencing method identifying only six samples. Almost 83% of isolates belonged to the class Bacilli (phylum Firmicutes) and another 13% were identified as belonging to *Desulprotobacteri*a. The percent identity calculated from the results describes the similarity between the query and target sequences. *Bacillus amyloliquefaciens* gives the highest percent identity, which is 98.85% similar to the target sequence. The higher the percent identity is, the more significant the match.

Table 3 shows the morphological and physiological characteristics of the six identified bacteria from the rice straw burning activities. The description of morphological and physiological characteristics was based on the previous study observation.

## 4. Discussion

The PM_2.5_ recorded in this study exceeded the limit suggested by MAAQS-2020 and USEPA, which is 35 µg/m^3^, and measured the highest at 645 µg/m^3^. According to [8], assessing the effects of open rice straw burning on air quality is difficult because these fires typically occur infrequently, for short periods of time, and in small rice paddy plots spread across a large region [8]. The study in Thailand was conducted by [29] and found that PM_2.5_ concentrations ranged between 2–166 µg/m^3^ over a 24 h period (daily standard 50 µg/m^3^) and 9–36 µg/m^3^ over an annual average, bringing the national average to 22 µg/m^3^ (average standard 25 µg/m^3^). Furthermore, the number of days when PM_2.5_ concentrations exceeded the daily standard in Chiang Mai, a province in Thailand’s northern region where many biomass open burning activities can be found during the summer, was reduced from 57 to 29 g/m^3^ in 2016 compared to 2017. The annual average of PM_2.5_ was 30 µg/m^3^ in 2017.

The morphological analysis of PM_2.5_ conducted using FESEM-EDX showed that the collected particles (no larger than 2.5 µm) mainly had a chain-like and irregular shape. Chain-like particles are soot aggregates resulting from biomass and combustion, as well as gasoline and diesel exhaust emissions [30,31]. Mineral matter from agricultural dust, windblown dust, resuspended road, and construction dust comprise the majority of irregular particles [30,32].

According to a study based on ICP-OES analytic results [33], potassium was the main ingredient, comprising 7.2 percent for wheat straw and 6.9 percent for maize stover. However, from this study, we found the average concentration of Ca to be the highest. The concentration of K discovered in this study was quite high, which cannot be ignored in terms of its effect on human health.

*Exiguobacterium* species are voluntary anaerobic, non-spore-forming, Gram-positive bacilli, rarely associated with human infections. A study conducted by [34] observed that the pathogenic potential of the *Exiguobacterium genus* is emphasized, because of its uniqueness and its clinical importance, which codes for different virulence factors and those associated with antibiotic resistance. According to the findings, the *Exiguobacterium sp. AT1b/GX59* strain has a number of factors that help it adapt to a pathogenic lifestyle, including hemolysin, secretion systems, chemotaxis proteins, and antibiotic resistance genes [34]. *Exiguobacterium* is a diverse genus with remarkable adaptability to a wide range of extreme conditions, which may be a valuable resource for developing environmentally friendly biologic alternatives to reduce chemical-intensive farming practices and improve long-term agricultural productivity [35].

Bacillus species, such as *B. amyloliquefaciens*, are widespread in soils, including agricultural settings, and are naturally present in fresh foods. *Bacillus amyloliquefaciens* is unrelated to the human pathogen species *Bacillus anthracis* and *Bacillus cereus*, which are both members of the *Bacillus cereus* community. Bacillus amyloliquefaciens is a member of the *B. subtilis* genus, which is a somewhat homogeneous group. For many years, different *B. subtilis* species, especially *B. subtilis* and *B. amyloliquefaciens*, have been used in biotechnology to produce enzymes, surfactants, probiotics, and antibiotics. *B. cereus* consists of Gram-positive spore-forming bacteria that produce toxins linked to foodborne illnesses. *B. proteolyticus* were proposed as new members of the *B. cereus* group [36].

Lysinibacillus, a recently reclassified genus of Bacillus, has been confirmed to have the ability to control pests, remediate heavy metal-contaminated habitats, and boost crop yields [37]. Lysinibacillus spp. that are Zn-tolerant have also been documented to promote maize growth in Zn-contaminated soil [37].

From this research, the most abundant bacteria found from rice straw burning activities was found to be a genus of Gram-positive bacteria. Based on this current study, the species of bacteria from rice straw burning activities was successfully found.

## 5. Conclusions

PM is a major pollutant, and its concentration is typically higher during agricultural burning activities than during non-burning activities. The result shows that the 12 h mean average concentration ± standard deviation of PM_2.5_ at S1 (BB) (41.00 ± 58.82 µg/m^3^) and at S2 (BB) (65.33 ± 175.10 µg/m^3^) exceeded the 35 µg/m^3^ limit suggested by USEPA and MAAQS-2020. Natural particles and anthropogenic particles categories were found in this study. The mean concentration of Ca element in PM_2.5_ was found to be the highest among other elements during rice straw burning activities at S2 followed by the concentration of Na.

The species identified from this study were Exiguobavterium indicum, Bacillus amyloliquefaciens, Desulfonema limicola str. Jadabusan, Exiguobacterium acetylicum, Lysinibacillus macrolides, and Bacillus proteolyticus. The species were identified based on the similarity between biological sequences using BLAST (Basic Local Alignment Search Tool) provided by NCBI (National Center for Biotechnology Information). *B. proteolyticus* should be considered a pathogenic bacteria as it is classified as a member of the *B. cereus* group. However, based on this research, not all the bacteria could be identified using the method approached. Therefore, further study should be done to obtain other species of bacteria from the polluted air from the rice straw burning activity, which may impact the health of humans.

In conclusion, this study demonstrates ad hoc concentration of PM_2.5_ that occurred only for a short period of time during rice straw burning activity. However, the results obtained from this study proved that the higher concentration of PM_2.5_ affecting the concentration of trace element concentration depends on the location of the sampling area and meteorological components. *Bacillus sp*. was found to be the most abundant in this study from the application of culturable method.

## Figures and Tables

**Table 1:** Variation of PM_2.5_ concentration and meteorological parameters monitored during background (B) (*N* = 1440) and biomass burning (BB) (*N* = 720) at Site 1 and Site 2.

Parameter	Site Category	Mean	Median	Standard Deviation	Minimum	Maximum
PM_2.5_ Conc. (µg/m^3^)	S1 (B)	9.79	9.00	6.39	1	28
	S1 (BB)	41.00	21.50	58.82	10	233
	S2 (B)	14.79	15	7.98	4	40
	S2 (BB)	65.33	9.5	175.10	4	645
T (°C)	S1 (B)	30.34	31.20	2.67	23.70	34.60
	S1 (BB)	30.61	30.35	2.73	24.20	35.50
	S2 (B)	29.30	28.90	2.97	24.00	35.80
	S2 (BB)	28.88	28.90	2.71	23.80	33.40
RH (%)	S1 (B)	62.23	59.00	11.21	44	84
	S1 (BB)	60.59	61.00	12.75	39	84
	S2 (B)	67.95	67.00	11.93	45	88
	S2 (BB)	67.44	67.50	9.97	50	86
WS (m/s)	S1 (B)	0.81	0.40	0.69	0.30	3.30
	S1 (BB)	0.64	0.40	0.50	0.30	2.70
	S2 (B)	0.34	0.30	0.18	0.30	2.00
	S2 (BB)	0.47	0.30	0.38	0.30	2.50

**Table 2:** The result of bacteria identification based on Sanger sequencing and nucleotide sequence database using BLASTn program.

Sample	Bacteria Identified in the Sample	Query Coverage (%)	Percent Identity (%)	Reference	NCBI Reference Sequence
1	Exiguobacterium indicum	76	81.29	Chaturvedi and Shivaji 2006	NR_042347
2	Bacillus amyloliquefaciens	99	98.85	Nishikawi et al. 2007	NR_041455
3	Desulfonema limicola str. Jadebusen	82	70.74	Fukui et al. 1999	NR_044782
4	Exiguobacterium acetylicum	99	98.81	Rodrigues et al. 2006	NR_043479
5	Lysinibacillus macroides	99	98.55	Heryman et al. 2005	NR_114920
6	Bacillus proteolyticus	98	98.42	Liu et al. 2017	NR_157735

**Table 3:** Morphological and physiological characteristics of bacteria identified in the sample.

Bacterial Identified in the Sample	Morphological Characteristics		Physiological Characteristics	Reference
	Colony Descriptions	Cell Features	Temperature Tolerance	
*Exiguobacterium indicum*	2–4 mm in diameter, round shape, yellowish orange, plating on nutrient agar plates and incubated at 22 °C for 3 days.	Gram-positive, motile, rod-shaped, non-spore-forming.	pH range: 6–10 (not grow at pH 4) Temperature range: 10–30 °C, optimum growth at 25 °C NaCl tolerance: 5–8%.	[23]
*Bacillus amyloliquefaciens*	Slightly raised, 0.58–0.65 × 92.7–3.9 µm in diameter, colonies were creamy-white and rough and grew rapidly on tryptic soy agar (TSA) at 30 °C after 24 h incubation.	Gram-positive, single, rod-shaped cells, slightly irregular.	N/A	[24]
*Desulfonema limicola str. Jadebusen*	Natural sea water medium was replaced with synthetic saltwater media. Grown at 28–30 °C	Gram-negative (although two strains-stained Gram-variable to Gram-positive Width x length of one cell (µm): (2.5–3 × 2.5–3.5), length of filaments (µm): 50–1000.	pH Range: 6.5–8.8, optimum at 7.6 Temperature range: 15–36 °C, optimum at 30 °C. The type of strain requires at least 12 g NaC1 and 2 g MgCl2 × 6H2O per 1 of culture medium for optimum growth and does not develop in freshwater medium.	[25]
*Exiguobacterium acetylicum*	Medium sized circular, slightly raised, yellow pigmented, smooth colonies of 4–6 mm diameter on nutrient agar at 15 °C after 3–4 days incubation	Gram-positive, short rods with scattered arrangement of cells.	pH range: 4 to 10 Temperature range: 4 to 42 °C, optimum 30 °C NaCl tolerance: 2 to 8%	[26]
*Lysinibacillus macroides*	Moist and loose texture, circular shape, cream colored, with irregular edges and glossy surfaces. 24–48 h incubation at 30 °C on TSA containing 5mg MnSO4 1-1, diameter after 24 h incubation 0.5–1.0 mm; reach 3.0–5.0 mm after 48 h.	Gram-positive and Gram-negative motile rod, spore-forming bacterium, Cell length 0.9–1.1 × 3.0–5.0 µm on plate cultures, but long filaments of 10–100 µm or more may be formed in broth cultures.	pH range: 7.0–9.0, optimum pH 8.0. Not growth at pH 6.0. Temperature range: 20 °C to 45 °C. NaCl tolerance: In the presence of 0% (*w*/*v*) NaCl and in up to 4% (*w*/*v*) NaCl, but not in 5% (*w*/*v*).	[27]
*Bacillus proteolyticus*	Milk white, circular, non-translucent, 2–3 mm in diameter after incubation at 32 °C for 48 h on Luria-Bertani medium	Gram-positive, anaerobic, and non-motile, rod shape, central elliptical endospore, 1.6–1.8 µm in width and 2.8–3.6 µm in length.	pH range: 5–10, optimum pH8 Temperature range: 10–39 °C, optimum 30 °C. NaCl tolerance: 0–9% (*w*/*v*), optimum 0–1%.	[28]
